# Coronary atherosclerosis in middle‐aged athletes: Current insights, burning questions, and future perspectives

**DOI:** 10.1002/clc.23340

**Published:** 2020-02-07

**Authors:** Vincent L. Aengevaeren, Thijs M. H. Eijsvogels

**Affiliations:** ^1^ Department of Physiology Radboud Institute for Health Sciences, Radboud University Medical Center Nijmegen The Netherlands; ^2^ Department of Cardiology Radboud Institute for Health Sciences, Radboud University Medical Center Nijmegen The Netherlands

**Keywords:** atherosclerosis, exercise testing and exercise physiology, computed tomography imaging

## Abstract

Regular exercise training is considered healthy as it reduces the risk of cardiovascular events and mortality. Nevertheless, athletes are not immune to the development of cardiovascular diseases and recent studies reported a higher prevalence of coronary artery calcifications and atherosclerotic plaques in athletes compared to less active controls. These observations have raised many questions among sport scientists, sports cardiologists, amateur athletes, and the general population. For example, Are athletes (not) immune for coronary atherosclerosis? How to assess coronary atherosclerosis in athletes? What about chalk (calcified plaque) and cheese (mixed plaque)? Does exercise intensity play a role? Are there sport‐related differences? Are there sex differences? Can sports medical evaluation detect coronary atherosclerosis? Do athletes get worried? Should athletes get worried? How should athletes with coronary atherosclerosis be managed? The goal of this review is to discuss the latest scientific insights and to answer these important questions. Furthermore, we will explore potential clinical implications and point out directions for further research.

## 1 INTRODUCTION

“If we could give every individual the right amount of nourishment and exercise, not too little and not too much, we would have found the safest way to health”

Hippocrates, 460‐377 bc

It has been known for more than 2000 years that habitual physical activity and regular exercise training importantly contribute to a healthy lifestyle. Sufficient physical activity and exercise are associated with remarkable reductions in cardiovascular events^1^, ^2^, ^3^, ^4^, ^5^ and increased longevity.^6^ Only 15 minutes of physical activity per day is associated with a 14% reduction in all‐cause mortality.^4^ In contrast, insufficient physical activity is responsible for a population attributable fraction of 12% for cardiovascular disease (CVD) mortality.^7^ As such, exercise is one of the most effective measures to reduce the risk of CVD in general, and coronary heart diseases in particular as ≥44% of CVD deaths are attributable to atherosclerotic coronary heart disease. Exercise‐induced CVD risk reductions do, therefore, translate to large health benefits as CVDs are the primary cause of death worldwide, accounting for ±18 million deaths per year (±31% of total mortality).^7^

The common dogma is that more exercise yields greater health benefits. However, recent studies reported intriguing findings on the effects of long‐term high‐volume high‐intensity exercise training on the prevalence and severity of atherosclerotic coronary heart disease among amateur athletes.^8^, ^9^, ^10^ A higher prevalence of coronary artery calcifications (CACs) and atherosclerotic plaques has been found in athletes vs controls, with a higher CAC and plaque prevalence in the most active vs least active athletes. These findings have raised many questions among healthcare professionals, sports scientists, and athletes. The goal of the present review is to discuss the latest insights from this field of research and to provide evidence‐based answers to these eminent questions.

## 2 ARE ATHLETES (NOT) IMMUNE TO CORONARY ATHEROSCLEROSIS?

The development and progression of CVD is importantly dependent on lifestyle factors, including physical activity and nutrition.^7^ It has been suggested that atherosclerosis is typically a disease of modern society, as physical inactivity and atherogenic diets are common nowadays. However, when researchers made computed tomography (CT) scans of mummies from ancient Egypt, 20 out of 44 mummies (45%) had atherosclerosis at an estimated age of 45 ± 9 years.^11^ Of those mummies, two had definite coronary atherosclerosis, the earliest documentation of coronary atherosclerosis. A postmortem pathology study from 1960 investigated coronary atherosclerosis in men aged 30 to 60 years who died suddenly from accidents, homicide, or suicide.^12^ Study participants were classified as sedentary (eg, accountant, bank clerk, and chauffeur) or physically active (eg, construction worker, gardener, and plumber) based on their occupation history. Surprisingly, a similar degree of coronary atherosclerosis was found among the sedentary and physically active group, suggesting that development of coronary atherosclerosis was not associated with habitual physical activity.^12^ In 1977, Bassler described a case series of runners who had died.^13^ Interestingly, autopsy reports revealed that none of the marathon runners died of atherosclerotic coronary artery disease, suggesting that marathon runners could be immune for coronary atherosclerosis as they avoided tobacco smoking and unhealthy diets and covered great distances on foot.^13^ Unfortunately, a 1979 case series of four marathon runners reported autopsy‐proven coronary atherosclerosis, which rejected the Bassler's hypothesis that long‐distance runners are protected from the development of coronary heart disease.^14^ In conclusion, the development of atherosclerosis is importantly dependent on lifestyle factors such as insufficient physical activity and an atherogenic diet, but athletes are not immune to atherosclerosis.

## 3 HOW TO ASSESS CORONARY ATHEROSCLEROSIS IN ATHLETES?

Although older studies relied on autopsy to determine the presence and severity of coronary atherosclerosis, the development of imaging techniques changed the availability of methods to assess coronary heart disease characteristics. Nowadays, coronary atherosclerosis is typically measured non‐invasively using a CT scan. This can be done with or without a contrast agent. A non‐contrast CT scan allows quantification of CAC by calculating a CAC score (CACS).^15^ The Agatston CACS is calculated by multiplying the area of each calcification by 1, 2, 3, or 4 depending on the density of the area, and summing up the scores for all slices. The density score is based on the highest Hounsfield units (HU) of the area, with a density score of 1 for HU 130 to 199, 2 for 200 to 299, 3 for 300 to 399, and 4 for ≥400 HU.^16^ CAC scoring only includes areas with a density ≥130 HU and ≥1 mm^2^. The CACS is related to the coronary atherosclerotic burden and a strong predictor for future CVD.^17^ In a study of 4425 patients followed up for 3 years, the probability of major adverse cardiac events was estimated for CACS categories. Patients without CAC (a CACS of 0) had a major adverse cardiac event rate of 2.1%. The rate of cardiac events was 12.9% for those with CACS >0 to 100, 16.3% for those with CACS >100 to 400, and 33.8% for those with CACS >400, indicating the strong prognostic value of CAC.^18^ As such, CACS are widely used to assess atherosclerotic burden and future cardiovascular risk in an easy, non‐invasive way. Interestingly, although the CACS itself is a multiplication of CAC area and density, a distinction can be made between the CV risk associated with increases in the area and density of CAC regions. Higher CAC area (or volume) is associated with a higher CVD risk, whereas a higher CAC density is associated with a lower risk of CVD.^19^

Using a contrast‐enhanced coronary CT scan (coronary CT angiography, CCTA), the lumen of the coronary arteries can also be imaged and plaque characteristics can be determined. This allows for determination of plaque morphology and divide plaques into calcified, non‐calcified, and mixed (both calcified and non‐calcified parts) plaques.^20^ The differentiation of plaques based on their morphology into calcified, non‐calcified, and mixed plaques importantly impacts the associated CV risk.^18^ For example, in the previously mentioned study of 4425 patients, calcified plaques were associated with a 3‐year major adverse cardiac event risk of 5.5%, whereas this was 22.7% for non‐calcified plaques and 37.7% for mixed plaques.^18^ As CAC scoring only includes plaques with an area of density ≥130 HU and ≥1 mm^2^, plaque components below that threshold are not included in the CACS. This means that non‐calcified or only minimally calcified, mixed plaque (“cheese”) can only be detected using a contrast‐enhanced CT. Moreover, CCTA also allows for assessment of high‐risk plaque features such as the napkin‐ring sign, positive remodeling, low‐attenuation (<30 HU) plaque, and spotty calcification, which are also associated with worse prognosis.^21^

CT scanning uses radiation to construct images and tremendous efforts have been made over the last decades to reduce the radiation dose associated with CT scanning. A routine CCTA examination can now be performed at 2 to 4 mSv, while newer technologies even allow CCTA acquisitions at <1 mSv.^22^ CAC scoring also requires ±1 mSv of radiation, but recent efforts suggest CAC scoring can be improved and done at lower doses requiring ±0.2 to 0.3 mSv of radiation.^23^ Although the associated radiation dose is not high, it is not negligible and thus performing CAC scoring or CCTA should clearly be of value to the individual.

## 4 DO CORONARY ATHEROSCLEROSIS CHARACTERISTICS DIFFER BETWEEN ATHLETES AND CONTROLS?

Although several studies demonstrated that athletes are not immune to coronary atherosclerosis, it was generally assumed that coronary atherosclerosis would be less prevalent in athletes compared to the general population. In 2008, Möhlenkamp et al studied 108 male marathon runners and compared them to 864 age‐matched and 216 age‐ and risk factor‐matched control subjects from the Heinz‐Nixdorf Recall study.^10^ They found that the German marathon runners had similar CACS compared to age‐matched controls, but higher CACS compared to the controls matched for both age and risk factors (Table 1). These findings were surprising and suggested more coronary atherosclerosis in athletes vs controls. Criticists raised the possibility that the inclusion criteria may have caused bias. Participants older than 50 years with ≥5 marathon completion in the previous 3 years were recruited for the study. Hence, marathoners might have relatively recently adopted an active lifestyle, which improved their risk factors whereas their CACS reflected their prior exposure to higher risk factors. Nevertheless, Merghani et al confirmed findings from the German study and reported that male British athletes had higher CACS than control subjects (median CACS of 86 vs 3, *P* = .02), but only in those with prevalent CAC^9^ (Table 1). Similarly, Aengevaeren et al found among 284 Dutch male athletes that the most active athletes (>2000 metabolic equivalent of task [MET]‐min/wk) more often had CAC (68%, adjusted odds ratio [OR_adjusted_]: 3.2; 95% confidence interval [CI]: 1.6‐6.6) compared with the least active athletes (<1000 MET‐min/wk, 43%).^8^ However, CACS did not differ between the athletes with prevalent CAC in their study. DeFina et al studied 21 758 generally healthy American men and divided them based on their physical activity level into individuals performing <1500, 1500 to 2999, and ≥ 3000 MET‐min/wk.^24^ The most active individuals were more likely to have a CACS >100 (OR_adjusted_: 1.11; 95% CI: 1.03‐1.20) compared with individuals performing less physical activity. Collectively, these findings indicate that athletes are more likely to have high CACS than controls.

**Table 1:** Coronary atherosclerosis characteristics across studies comparing athletes and controls

	Möhlenkamp (2008)^10^			Merghani (2017)^9^		Aengevaeren (2017)^8^		DeFina (2019)^24^	
Exercise volume group	Marathon runners (n = 108)	Age‐matched controls (n = 864)	Age and RF‐matched controls (n = 216)	Athletes (n = 106)	Controls (n = 54)	Most active (>2000 MET‐min/wk) (n = 75)	Least active (<1000 MET‐min/wk) (n = 88)	Most active (>3000 MET‐min/wk) (n = 1561)	Less active (<3000 MET‐min/wk) (n = 20 197)
CAC prevalence (%)	71	82^a^	69	48	41	68	43^a^	NA	NA
CAC prevalence (OR)	NA	NA	NA	NA	NA	3.2 (1.6–6.6)	1.00 (reference)^a^	1.11 (1.03‐2.20)	1.00 (reference)^a^
CACS in all individuals (AU)	36 (0‐217)	38 (3‐187)	12 (0–78)^a^	0	0	9.4 (0‐60.9)	0 (0‐43.5)^a^	NA	NA
CACS in CAC >0^b^ (AU)	NA	NA	NA	86	3^a^	39 (8‐159)	69.6 (14‐332)	NA	NA
Plaque prevalence (%)	NA	NA	NA	44	22^a^	77%	56^a^	NA	NA

*Note*: Data shown as percentage or with 95% confidence interval or median (interquartile range).

Abbreviations: AU, Agatston units; CAC, coronary artery calcification; CACS, CAC score; MET, metabolic equivalent of task; NA, not available; OR, odds ratio; RF, risk factor.

a Significantly (*P* < .05) different from athletes/most active cohort.

b CACS only in CAC > 0 thus lower number of participants.

Aengevaeren et al and Merghani et al also performed a contrast‐enhanced CT scan following CAC scoring. Prevalence of atherosclerotic plaques was higher in male athletes compared to controls (44% vs 22%)^9^ and in the most active athletes (77%; OR_adjusted_: 3.3; 95% CI: 1.6‐7.1) compared to the least active athletes (56%).^8^ However, plaque morphology differed significantly across groups. Athletes demonstrated predominantly calcified plaques (73%) instead of mixed plaques (23%), whereas controls had fewer calcified plaques (31%, *P* = .0006) and predominantly mixed plaques (62%, *P* = .0002).^9^ Similarly, the most active athletes had less often atherosclerotic plaques of a mixed morphology (48%; ORadjusted: 0.35; 95% CI: 0.15‐0.85) compared with the least active athletes (69%). The most active athletes also had more often only calcified plaques compared to the least active athletes (38% vs 16%; OR_adjusted_: 3.57; 95% CI: 1.28‐9.97). These findings indicate a more benign plaque composition in athletes vs controls, as calcified plaques are associated with a lower cardiovascular risk than mixed plaques.^18^ The comparison of atherosclerosis induced health risks between athletes and the general population is therefore inappropriate, as “chalk” (ie, low‐risk calcified plaques) has different risk estimates compared to cheese (ie, higher risk non‐calcified/mixed plaques). These observations emphasize that personalized medicine is needed in the treatment of coronary atherosclerosis.

## 5 DOES EXERCISE INTENSITY PLAY A ROLE?

Exercise encompasses a wide subset of activities that can be performed at a different frequency, duration, and intensity. Little is known about the influence of exercise intensity on coronary atherosclerosis. Aengevaeren et al explored the association between different exercise intensities and found that only very vigorous intensity exercise (≥9 MET) was significantly associated with increased CAC (OR_adjusted_: 1.47; 95% CI: 1.14‐1.91) and plaque (OR_adjusted_: 1.56; 95% CI: 1.17‐2.08).^8^ Follow‐up studies are needed to confirm these initial findings, whereas mechanistic studies may unravel how and why higher exercise intensities are associated with more coronary atherosclerosis.

## 6 ARE THERE SPORT‐RELATED DIFFERENCES?

**Figure 1:**
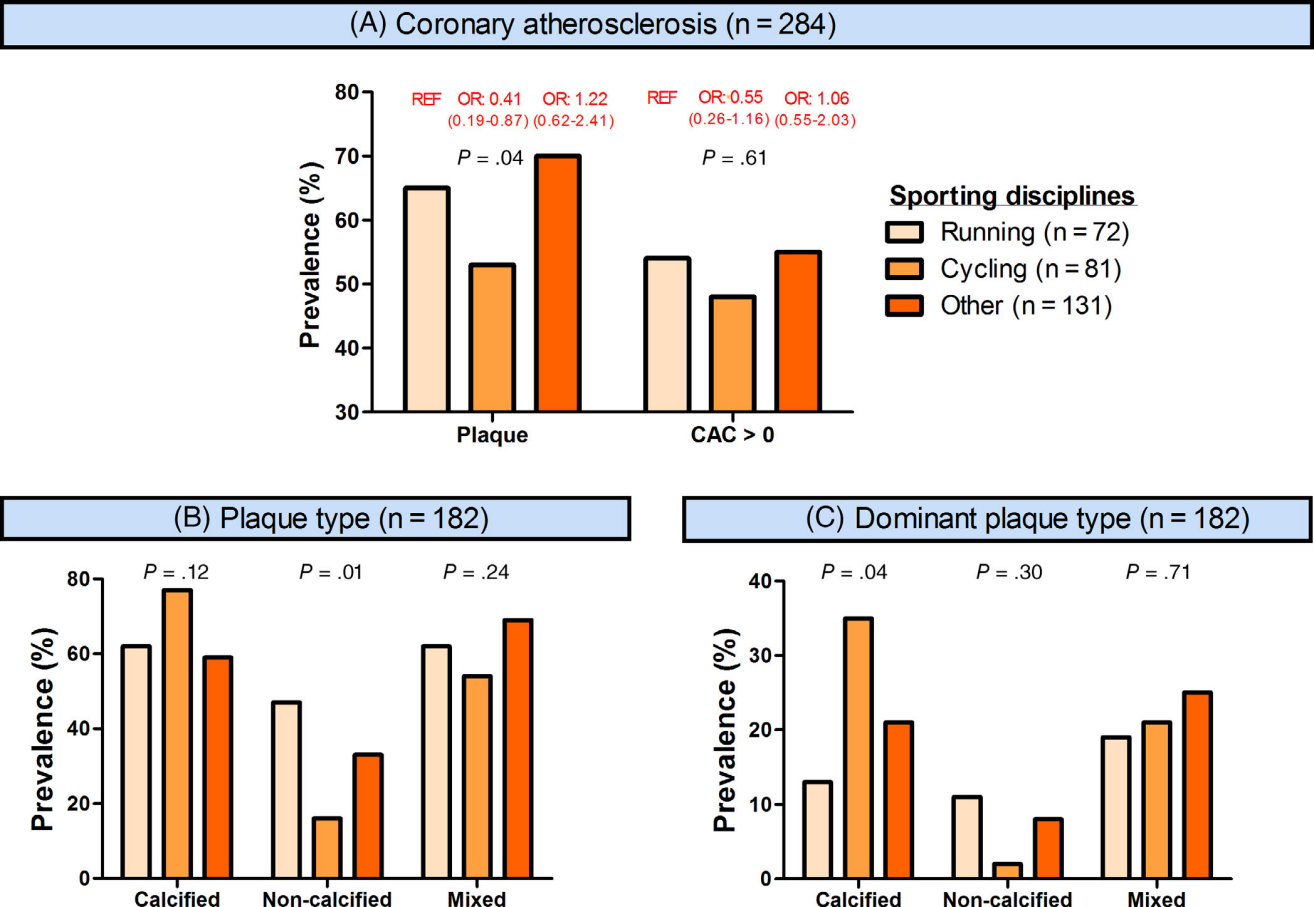
Prevalence of coronary atherosclerosis and plaque morphology across sporting disciplines in participants from the Measuring Athletes' Risk of Cardiac events (MARC) study. CAC, coronary artery calcification; OR, odds ratio; REF, reference

Most studies assessing coronary atherosclerosis in middle‐aged athletes recruited predominantly runners and cyclists. Aengevaeren et al investigated whether there were differences between runners (n = 72, 25%), cyclists (n = 81, 29%), and athletes performing other types of sport (n = 131, eg, soccer [n = 29], hockey [n = 11], and water polo [n = 10]). They found that cyclists had a lower prevalence of atherosclerotic plaques (OR_adjusted_: 0.41; 95% CI: 0.19‐0.87) and a trend toward lower prevalence of CAC (OR_adjusted_: 0.55; 95% CI: 0.26‐1.16) compared with runners (Figure 1). Moreover, they found that among athletes with plaques, cyclists had a somewhat more benign atherosclerotic plaque composition with a similar prevalence of mixed plaques (OR_adjusted_: 0.78; 95% CI: 0.31‐1.94) and more often only calcified plaques (OR_adjusted_: 3.59; 95% CI: 1.14‐11.31) compared with runners.^25^ Merghani et al also compared male runners (n = 82) and cyclists (n = 24), and found no significant differences with respect to CAC prevalence (50% vs 42%), CACS >100 (17% vs 25%), CACS >70th percentile (16% vs 13%), or a luminal stenosis ≥50% (8.5% vs 4.2%). A potential explanation for the different outcomes across studies may relate to the relatively low sample size of subgroups. Moreover, athletes may perform more than one sport, thereby making the impact of a single sport difficult and uncertain to elucidate.

Future studies with larger populations are needed to provide definitive answers to sport‐related differences, whereas inclusion of athletes performing other types of sports (ie, swimming, football, soccer, etc.) are also warranted.

## 7 ARE THERE SEX DIFFERENCES?

The large majority of participants in the current studies have been males. This is mostly due to the fact that males have a higher prevalence of coronary atherosclerosis and a higher risk of sudden cardiac death during exercise.^26^, ^27^ However, there have been several studies investigating female athletes. Merghani et al included 46 female athletes and 38 female controls.^9^ They found no differences in CAC prevalence (22% vs 32%, *P* = .33), CACS ≥100 (7% vs 11%, *P* = .62), or plaque prevalence (15% vs 21%, *P* = .57). The authors indicated that this might have been due to a relatively low power due to the inclusion of premenopausal women who are relatively protected from coronary atherosclerosis. Roberts et al studied 26 female marathon runners and compared them to 28 sedentary women referred for CCTA to evaluate coronary artery disease.^28^ The authors found a lower plaque prevalence (19% vs 50%, *P* = .01) in the athletic women. However, the control subjects had a significantly higher body mass index as well as a higher prevalence of hypertension, hyperlipidemia, smoking history, and family history for coronary artery disease, questioning the validity of this comparison. DeFina et al found no association between physical activity categories and prevalence of CACS ≥100 in female participants, whereas this association was present for men.^24^ Although limited evidence is available, current data suggest that the association between exercise and coronary atherosclerosis is weaker in female athletes compared to their male counterparts, which may be mediated by estrogen.

## 8 CAN SPORTS MEDICAL EVALUATION DETECT CORONARY ATHEROSCLEROSIS?

**Figure 2:**
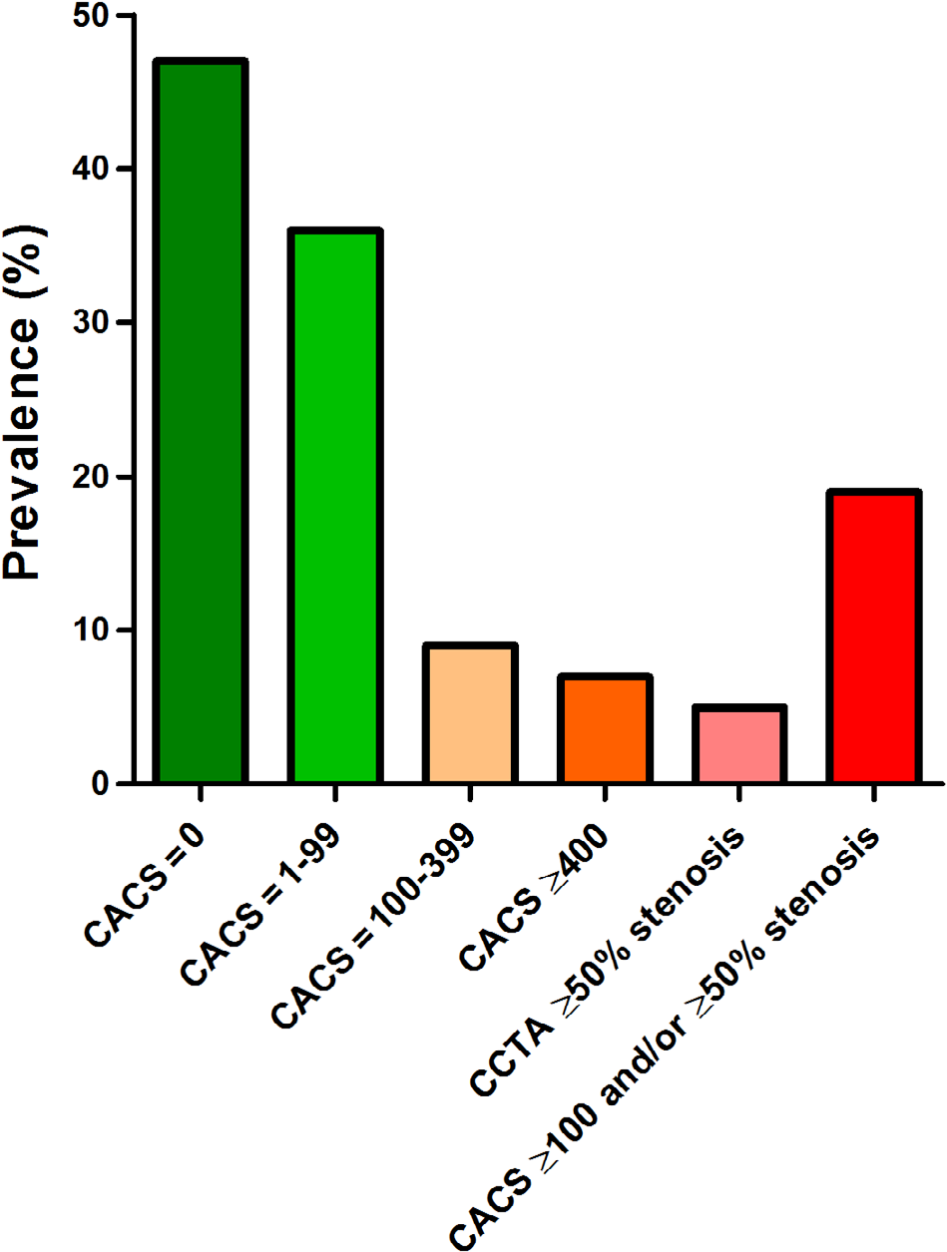
Prevalence of CACS and obstructive (>50%) coronary artery disease in participants from the Measuring Athletes' Risk of Cardiac events (MARC) study. CACS, coronary artery calcification score; CCTA, coronary computed tomography angiography

A sports medical evaluation typically includes taking a medical history, physical examination, resting electrocardiogram, and bicycle exercise test with electrocardiogram. However, Braber et al previously showed that among 318 middle‐aged male amateur athletes who underwent a sports medical evaluation without abnormalities, as much as 19% had a CACS ≥100 and/or ≥50% stenosis^29^ (Figure 2). These findings suggest that a substantial proportion of asymptomatic athletes can have subclinical atherosclerosis that goes undetected during sports medical evaluations and may place them at risk of (exercise‐induced) cardiac events.

Braber et al also calculated the number needed to screen with CAC scoring and with CAC scoring plus CCTA to prevent one cardiovascular event (ie, angina pectoris, myocardial infarction, coronary revascularization, resuscitated cardiac arrest, stroke, or cardiovascular death) within 5 years.^29^ The estimated number needed to screen using only CAC scoring was 183 (95% CI: 144‐236) and 159 (95% CI: 128‐201) for CAC scoring plus CCTA. Although it is currently not recommended to implement CAC screening programs among middle‐aged and older athletes, one could argue that this might become an option in the future considering the ongoing debate on improving cardiovascular screening of athletes and especially given the high negative predictive value of a CACS of zero.^30^, ^31^

## 9 DO ATHLETES GET PSYCHOLOGICAL STRESS FOLLOWING CORONARY CT?

CAC scoring is an increasingly popular diagnostic tool and many athletes have had CAC scoring, either for research or clinical purposes. Although CAC scoring provides a lot of valuable prognostic information, it may also increase psychological distress in athletes when either they have intermediate CACS not requiring treatment or when they have high(er) CACS requiring medication. If coronary CT is to be more widely implemented in the future, this is an important factor to take into consideration, especially in primary prevention. Schurink et al investigated the psychological distress in 275 athletes who underwent CAC scoring and CCTA for research purposes.^32^ They found that cardiovascular screening including a sports medical evaluation and cardiac CT did not increase overall psychological distress in a cohort of middle‐aged male amateur athletes. There was only one individual (1/275, 0.4%) who had experienced clinically relevant psychological distress following the examinations which showed substantial coronary artery disease, but overall the majority felt safer when exercising (59%), satisfied with their participation (94%), and would participate again (95%).^32^

## 10 SHOULD ATHLETES GET WORRIED ABOUT THEIR CARDIOVASCULAR RISK?

Although CAC is associated with increased cardiovascular risk, DeFina et al showed that among individuals with CACS <100 all‐cause mortality was lower in the most active individuals (≥3000 MET‐min/wk, hazard ratio [HR]: 0.52; 95% CI: 0.29‐0.91) compared with the least active individuals (<1500 MET‐min/wk), whereas among individuals with CACS >100 the risk was not significantly different between physical activity volume groups (HR: 0.77; 95% CI: 0.52‐1.15).^24^ Similarly, Arnson et al studied the impact of self‐reported exercise on the relationship between CACS and mortality among asymptomatic patients.^33^ Patients were asked: “On a scale of 0 to 10, how much do you exercise (0—none, 10—always)?” This question was then categorized as “no exercise” (score of 0 or 1), “low exercise” (score of 2‐5), “moderately active” (score of 6‐8), and “highly active” (score of 9 and 10). With the highly active as reference group, being moderately active had an HR of 1.29 (95% CI: 0.86‐1.95) for all‐cause mortality, low exercise had an HR of 1.56 (95% CI: 1.06‐2.30), and no exercise had an HR of 2.35 (95% CI: 1.49‐3.70) after adjusting for CACS and potential confounders. These data indicate that individuals with substantial CAC who perform high volumes of exercise have a lower mortality risk than their less active and inactive peers.

Data from epidemiological studies show a higher life expectancy in athletes compared to less physically active control subjects. For example, both elite^34^, ^35^ and amateur^6^ athletes live longer than the general population. Exercise training increases longevity by approximately 3 years with the most benefit for endurance sports.^6^, ^35^ The lower risk of a given CACS and increased longevity in athletes may be explained by other beneficial coronary adaptations to exercise, such as increased epicardial coronary diameter, capillary density, and function.^36^, ^37^, ^38^ Hence, athletes should not get worried about the long‐term risks of potential detrimental adaptations to the athletes heart since they live ±3 years longer than the general population.

## 11 HOW SHOULD ATHLETES WITH CORONARY ATHEROSCLEROSIS BE MANAGED?

Treatment depends on the athlete's risk of CVD. Athletes should be questioned about cardiac symptoms, risk factors, and family history of CVD. Athletes may present with atypical symptoms such as reduced exercise performance, shortness of breath, or fatigue instead of typical angina. Athletes with cardiac symptoms should be investigated and managed as the general population.

**Figure 3:**
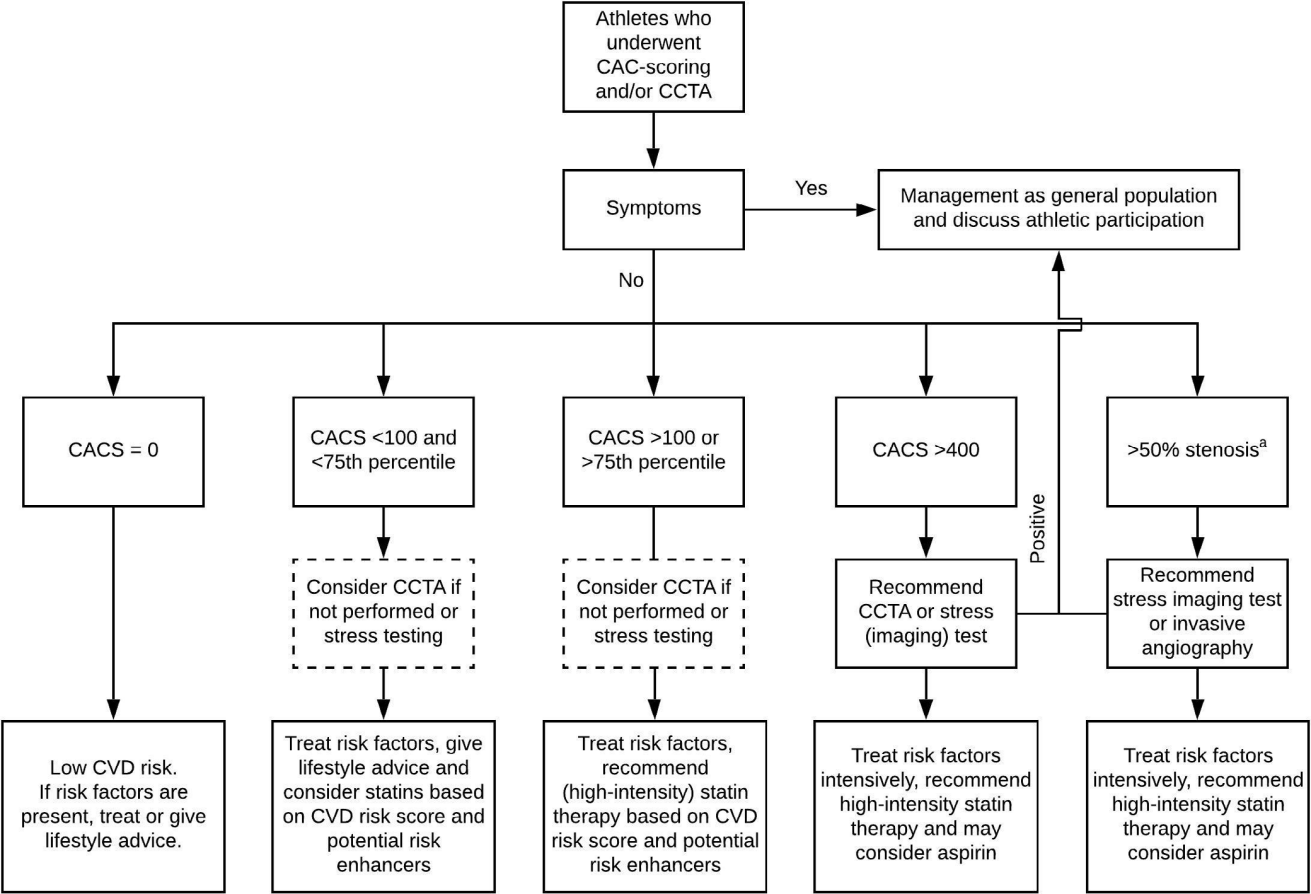
Flowchart for clinical management of athletes who underwent CAC scoring and/or CCTA. CAC, coronary artery calcification; CACS, CAC score; CCTA, coronary computed tomography angiography; CVD, cardiovascular disease. ^a^If CCTA was performed

There are no specific guidelines for the management of asymptomatic athletes with coronary atherosclerosis, but we have recently suggested a potential management strategy.^39^ In short, cholesterol‐lowering therapy should be advised according to recent guidelines,^30^, ^40^ taking into account, among others, the athlete's preferences concerning medication, their family history, and non‐calcified plaque on CCTA. If athletes have high CACS (≥400), they should be advised to start high‐intensity statin therapy regardless of CVD risk score and be informed to strictly control their cardiovascular risk factors (eg, blood pressure, glucose level, and smoking). Although its use in primary prevention has recently been debated, perhaps aspirin can be considered for individuals with (very) high CACS who are not at increased bleeding risk.^41^ Subsequent anatomical or functional testing is heavily dependent on local availability and costs of different tests. CCTA can be considered as an anatomical test to evaluate coronary stenoses, plaque morphology, and number and location of plaques. Stress (imaging) tests can be used as a functional measure of inducible ischemia. We suggest that for athletes with CACS ≥400, a CCTA to evaluate luminal narrowing or stress (imaging) test to evaluate potential ischemia should be considered, whereas for athletes with luminal stenoses >50%, a stress (imaging) test should be considered to detect potential myocardial ischemia. These should not necessarily lead to coronary interventions, as there is currently no evidence that a stent will increase life expectancy in an asymptomatic athlete, but could provide maximal heart rate guidelines and guide training schedules and additional medical therapy in individuals with ischemia. Also, in certain cases, invasive coronary angiography with fractional flow reserve measurements can be considered based on the anatomical information from CCTA. Figure 3 provides a basic schematic summary of these considerations.

Recommendations from the European Sports Cardiology Section for participation in leisure time or competitive sports for athletes‐patients with coronary artery disease provide guidance for participation in sports for athletes with asymptomatic coronary atherosclerosis.^42^ In short, athletes should be assessed for inducible ischemia and coronary risk factors. Exercise testing, potentially followed by stress imaging tests, to assess functional ischemia is advised and aggressive risk factor management according to the guidelines is important. In general, athletes may be advised to participate in all types of exercise if they have no evidence of inducible ischemia or arrhythmias and have a normal ejection fraction.^42^

## 12 REMAINING QUESTIONS AND FUTURE PERSPECTIVES

There are still several missing answers to questions regarding the relationship between exercise and coronary atherosclerosis, such as: How does coronary atherosclerosis progress over time in athletes? What are explanations for this association? What is the clinical relevance of increased coronary atherosclerosis in athletes?

Different international research groups are studying coronary atherosclerosis in athletes and this will provide more data in the coming years. The British research group (Merghani et al^9^) is currently increasing the size of their female cohort, providing more information on the relationship between exercise and coronary atherosclerosis among female athletes in the near future. The Dutch research group (Aengevaeren et al^8^) is currently performing a follow‐up study of their cohort, establishing the first longitudinal athletic cohort with contrast‐enhanced CT scans. This will provide more data on longitudinal effects of exercise on CAC and plaque (morphology). The American research group (DeFina et al^24^) is following a large cohort over time to investigate whether exercise volume is associated with the development of CAC. These studies together will provide new data to either strengthen or reject the current findings and will hopefully answer some of the remaining questions. The mechanisms explaining the findings of increased coronary atherosclerosis in athletes are uncertain and likely complex. The existing literature and studies cannot answer this question. Thus, animal studies and perhaps physiological clinical studies will likely be necessary to investigate which mechanistic pathways are responsible for these observations.

## 13 CONCLUSIONS

Exercise is healthy and substantially reduces the risk of cardiovascular events and mortality. However, recent studies suggest increased coronary atherosclerosis in athletes. The apparent paradox of increased coronary atherosclerosis despite lower cardiovascular risk and increased longevity in the (most active) athletes may be explained by the observations of more benign plaque morphology (chalk instead of cheese) in combination with beneficial exercise‐induced coronary adaptations. Future longitudinal studies are required to shed more light on potential mechanisms, clinical relevance, and optimal management of coronary atherosclerosis in athletes.

## CONFLICT OF INTEREST

The authors declare no potential conflict of interests.
